# Bacterial spectrum and antimicrobial resistance of cerebrospinal fluid pathogens in pediatric bacterial meningitis: a 7-year study in Southwest China with emphasis on post-neurosurgical cases

**DOI:** 10.3389/fcimb.2026.1842744

**Published:** 2026-07-10

**Authors:** Yanjun Wang, Fangling Dong, Cuifen Li, Qiyan Lv, Xiong Wu, Zhuo Kong, Honglin Liu

**Affiliations:** 1 Pediatric Intensive Care Unit, Kunming Children’s Hospital, Children’s Hospital Affiliated to Kunming Medical University, Kunming, Yunnan, China; 2 Pediatrics Department of Yuanjiang County Hani, Yi and Dai Autonomous County People’s Hospital, Yuxi, Yunnan, China; 3 Department of emergency, Kunming Children’s Hospital, Children’s Hospital Affiliated to Kunming Medical University, Kunming, Yunnan, China

**Keywords:** antibiotic resistance, cerebrospinal fluid culture, pathogenic spectrum, pediatric bacterial meningitis, risk factors

## Abstract

**Background:**

The etiological profile and antimicrobial resistance (AMR) patterns of pediatric bacterial meningitis (PBM) are region-specific and dynamic. With limited data from Southwest China, this 7-year study aimed to characterize the pathogens and their AMR profiles to guide empirical therapy and infection control strategies.

**Methods:**

We retrospectively analyzed children diagnosed with PBM who had positive cerebrospinal fluid (CSF) cultures at Kunming Children’s hospital from September 2018 to September 2025. Clinical characteristics, laboratory findings, pathogen distribution, and antibiotic susceptibility were assessed. Risk factors for refractory meningitis and ICU admission were analyzed.

**Results:**

A total of 258 children (58.1% male; median age 17.0 months) were included. Gram-negative bacteria (GNB) accounted for 26.7% of cases. A significant portion (43.0%) had prior neurosurgery. Overall, 281 non-duplicate pathogens were isolated, with Gram-positive bacteria predominating (203/281, 72.2%) and Gram-negative accounting for the remaining 78 (27.8%). Overall, the three most common pathogens were coagulase-negative Staphylococci (CoNS) (37.0%, 104/281), *Streptococcus pneumoniae* (*S. pneumoniae*) (16.7%, 47/281), and *Escherichia coli* (*E. coli*) (12.5%, 35/281). Notably, pathogen distribution varied significantly with age: *E. coli* was predominant in infants (<12 months), while *S. pneumoniae* was more common in preschool children. Antimicrobial resistance was highly prevalent: The methicillin-resistant rate in CoNS was alarmingly high at 88.2%. Among GNB, fluoroquinolone resistance was the most dominant phenotype, detected in 74.3% of *E. coli* isolates. The detection rate of ESBL-producing *E. coli* fluctuated widely (0%-83.3%) during the study period. Difficult-to-treat resistance was less common, observed in 5.7% (2/35) of *E. coli* isolates. Complications including hydrocephalus (26.4%) and subdural effusion (24.4%) were frequent.

**Conclusion:**

In this cohort with a high proportion of post-neurosurgical cases (43.0%), CoNS was the most common pathogen (37.0%), with a methicillin resistance rate of 88.2%. Continuous and dynamic monitoring of the epidemic trends of pathogens and the resistance rates of antibacterial drugs is of vital importance for the treatment of PBM.

## Introduction

Pediatric bacterial meningitis (PBM) is a common acute central nervous system infectious disease and is one of the infectious diseases with a relatively high mortality rate, 30% to 50% survivors suffer from permanent neurological sequelae, with a prevalence rate of(6.95~22.30)/100–000 in children under five years old (Zainel et al., 2021; Snoek et al., 2022). The gold standard for diagnosis is cerebrospinal fluid (CSF) culture. Early diagnosis and targeted antibacterial treatment are of vital importance for improving patients’ survival rates, and preventing long-term neurological complications (Hasbun, 2022). However, when no clear pathogen can be identified, empirical treatment is the only clinical option. Therefore, understanding patients’ clinical features and the distribution of CSF pathogens and their resistance patterns to antibiotics holds significant clinical importance for the selection of empirical antibacterial drugs.

The epidemiological features of PBM are complex and variable, influenced by various factors such as the environment, region, and time. Previous studies have shown that the most common bacteria of PBM include *Streptococcus pneumoniae*, *Neisseria meningitidis* (*N. meningitidis*), and *Haemophilus influenzae*, whose incidence has decreased significantly worldwide with the use of vaccines (Li et al., 2018; Koelman et al., 2021). The misuse of broad-spectrum antibiotics has been a major factor in the escalating and alarming antimicrobial resistance (AMR) crisis. There are significant differences in the distribution of bacteria and drug resistance spectrum across different regions around the world (Awulachew et al., 2020; Ikken et al., 2020; Alhazmi et al., 2024; Dhraief et al., 2024; Mehmood et al., 2025). Even within China, the distribution of bacteria and resistance patterns vary from one region to another (Guo et al., 2016; Peng et al., 2021; Zhang et al., 2024a; Chen et al., 2025). Therefore, timely analysis and local reporting of the pathogenic factors and antimicrobial resistance patterns of PBM are essential to reinforce rather than redefine empirical therapy choices, by providing contemporary data on the resistance burden that clinicians face when following established guidelines.

Currently, there are limited reports in Southwest China focusing on the pathogen’s composition and resistance patterns of PBM over extended periods. In this study, we aimed to systematically and thoroughly analyze the composition and resistance patterns of bacteria isolated from CSF in Yunnan Province in the past seven years.

## Materials and methods

### Study design and patients’ inclusion

We conducted a retrospective cohort study including eligible children from Kunming Children’s Hospital from September 2018 to August 2025. The inclusion criteria were as follows: 1) patients aged 29 days to 14 years old; 2) meeting the PBM diagnostic criteria (Chen et al., 2025); 3) positive CSF culture. The exclusion criteria: 1) isolated organisms identified as contaminants or fixed values; 2) meningitis caused by fungi; 3) autoimmune neurological disorders; 4) primary immunodeficiency diseases; 5) incomplete clinical data. The flowchart was shown on **Supplementary Figure 1**. Of the 258 included patients, 111 (43.0%) had undergone neurosurgical procedures prior to meningitis onset, including external ventricular drain placement, ventriculoperitoneal shunt insertion, or craniotomy.

### Microbiology methods

For strain identification, we utilized the BD BACTEC FX 400 fully automatic bacterial culture system. The drug sensitivity tests were conducted using the VITEK2-CompAct fully automatic bacterial identification and drug sensitivity analyzer for routine minimum inhibitory concentration (MIC) tests. Cefoxitin disk diffusion (30 μg) was performed in coagulase-negative staphylococci according to *Clinical and Laboratory Standards Institute M100* (CLSI) guidelines. Isolates with a cefoxitin inhibition zone diameter <24 mm were classified as methicillin-resistant CoNS (MRCoNS). Briefly, a 30 μg cefoxitin disk was placed on Mueller-Hinton agar inoculated with a 0.5 McFarland standard suspension of the isolate. After 16–18 h of incubation at 35 °C, isolates with an inhibition zone diameter <24 mm was classified as MRCoNS. Susceptibility testing was performed using standard methods. The breakpoints for all antibiotics, except tigecycline, followed the guidelines in the annual editions (2018–2025) of the *CLSI M100*. For isolates tested in a given year, susceptibility breakpoints were applied according to the *CLSI M100* edition of that same year. No retrospective revision of breakpoints was performed for isolates tested in earlier years using newer CLSI editions. The European Committee on Antimicrobial Susceptibility Testing clinical breakpoints were applied for tigecycline. Isolates categorized as intermediate (I) were not categorized as resistant in the analysis.

The D-zone test was performed as recommended by CLSI for detection of inducible clindamycin resistance. We aimed to investigate the incidence rates of four major drug resistance phenotypes *among Gram-negative bacteria:* difficult-to-treat resistance (DTR), fluoroquinolone resistance (FQR), carbapenem resistance (CR), and extended-spectrum cephalosporin resistance (ECR). Definitions are as follows: DTR, the resistant or moderately resistant state to all β-lactam drugs, including carbapenems and fluoroquinolones. FQR, the resistance to ciprofloxacin or levofloxacin. CR, the resistance to imipenem or meropenem. ECR, the resistance to ceftriaxone or cefepime, but not including natural drug resistance.

### Data collection

We collected demographic and clinical data including age, gender, clinical symptoms, hematological index, CSF culture results, imaging data, and drug-resistance phenotypes. Comorbidities such as hydrocephalus, subdural effusion/empyema and cerebral abscess. Furthermore, data on the need for intravenous immunoglobulin (IVIG), corticosteroids and mechanical ventilation (MV) were also recorded. The length of hospital stays (LOS) was obtained.

### Definition and outcomes measurement

PBM with proven bacteria was defined as positive bacteria in the CSF culture with compatible symptoms and signs of meningitis (Guo et al., 2016). In this study, ICU admission was defined as transfer to the intensive care unit due to severe meningitis-related conditions (e.g., altered mental status, recurrent seizures, respiratory failure, septic shock, or need for vasopressor support). Patients who were admitted to the ICU solely for routine postoperative monitoring after neurosurgery (without active signs of severe meningitis) were not included in the ICU group for the purpose of this analysis. Refractory bacterial meningitis currently has no clear standardization. In our study, the definition was established based on previously published criteria. Pediatric patients meeting one or more of the following criteria were defined as refractory bacterial meningitis (Wu et al., 2023): 1) The duration of antibiotic use based on empirical or drug sensitivity tests has exceeded the standard of >14 days for GPB and >21 days for GNB, and there are still abnormal clinical manifestations, peripheral blood inflammatory indicators, and/or cerebrospinal fluid indicators. 2) Complications, such as subdural effusion, ependymitis, hydrocephalus, etc. 3) In the later stage of follow-up, death or sequelae occurred, such as secondary epilepsy, cranial nerve damage and/or psychomotor developmental dela. 4) There are recurrent bacterial infections of the brain that occur for unknown reasons. Community-acquired meningitis was defined as meningitis occurring in patients with no history of hospitalization or neurosurgical procedure within the 28 days prior to symptom onset. Hospital-acquired meningitis was defined as meningitis developing ≥48 hours after hospital admission, or within 7 days after discharge from a previous hospitalization, or in patients who had undergone neurosurgical procedures (including external ventricular drain, ventriculoperitoneal shunt, or craniotomy) during the current or prior admission. The main outcome of this study was the occurrence of ICU admission and refractory bacterial meningitis. The secondary outcomes included the length of hospital stay and the distribution of pathogens.

A contaminant was defined as the isolation of a potential skin commensal (e.g., CoNS, *Corynebacterium* spp., *Propionibacterium* spp., *Bacillus* spp. other than *B. anthracis*) from a single CSF culture in the absence of any clinical signs of meningitis (e.g., no fever, normal CSF parameters, or lack of antibiotic treatment). Isolates were also considered contaminants if they grew in mixed cultures with no clearly dominant organism and the patient’s clinical course did not suggest infection.

### Statistical analysis

Raw data was recorded in a secure electronic database in the form of a variable table. SPSS version 27.0 was used to perform statistical analyses. Quantitative data following normal distribution or not were denoted by mean ± standard deviation and median (IQR) values, respectively. Categorical variables were presented as frequencies (%). Quantitative data of the two groups were analyzed using the student’s t-test or Mann-Whitney U test, and categorical variables were analyzed using Chi-square or Fisher’s exact probability test. Logistic regression analyses were conducted to identify independent risk factors, and the results were reported as odds ratio (OR) and 95% confidence interval (CI). A difference was considered statistically significant when *P* was less than 0.05. For discrepancy in bacterial resistance patterns between different groups, only descriptive statistics (percentages, frequencies) were used; no hypothesis testing was performed.

## Results

### Description of study population and clinical features

During the study period, a total of 258 patients with PBM were identified, with a male percentage of 58.1% (150), an average age of 17.0 (4.8, 64.0) months and LOS of 28.0 (18.0, 41.0) days. Prior neurosurgery was present in 43.0% (111/258), and Gram-negative bacteria were isolated in 26.7% (69/258). Other complications included hydrocephalus (26.4%, 68/258), subdural effusion or empyema (24.4%, 63/258), and cerebral abscess (6.2%, 16/258). Demographic and clinical characteristics are summarized in **Supplementary Table 1**.

### Predictive risk factors for ICU admission and refractory meningitis

In multivariate analysis (**Table 1**), severe meningitis requiring ICU admission was associated with prior neurosurgery before diagnosis (OR = 0.488, 95% CI: 0.280-0.852, *P* = 0.012), Babinski sign (OR = 1.893, 95% CI: 1.056-3.394, *P* = 0.032), and albumin (OR = 0.937, 95% CI: 0.888-0.989, *P* = 0.019). Risk factors for refractory meningitis were age (OR = 0.993, 95% CI: 0.987-0.999, *P* = 0.039), prior neurosurgery (OR = 2.704, 95% CI: 1.559-4.691, *P* < 0.001), WBC >500× 10^6^/L in CSF (OR = 1.742, 95% CI: 1.004-3.022, *P* = 0.048) and the GNB detected in CSF culture (OR = 2.949, 95% CI: 1.517-5.732, *P* = 0.001).

**Table 1:** Predictive risk factors for ICU admision and refractory meningitis.

Variables	ICU admision				Refractory bacterial meningitis			
	Univariate analysis		Multivariate analysis		Univariate analysis		Multivariate analysis	
	OR (95% CI)	*P*	OR (95% CI)	*P*	OR (95%CI)	*P*	OR (95% CI)	*P*
Age	0.996 (0.991-1.002)	0.212			0.989 (0.984-0.995)	< 0.001*^c^	0.993 (0.987-0.999)	0.039*
LOS	0.990 (0.979-1.002)	0.103			1.020 (1.007-1.034)	0.002*^c^		
Surgery	0.473 (0.282-0.793)	0.005*^a^	0.488 (0.280-0.852)	0.012*	2.531 (1.521-4.210)	< 0.001*^d^	2.704 (1.559-4.691)	< 0.001*
Seizure	1.955 (1.083-3.528)	0.026*^a^			1.703 (0.936-3.099)	0.081		
Babinski sign	1.883 (1.107-3.204)	0.020*^a^	1.893 (1.056-3.394)	0.032*	1.106 (0.654-1.871)	0.706		
WBC	1.053 (1.018-1.091)	0.003*	1.034 (0.994-1.075)	0.100	1.002 (0.971-1.034)	0.887		
Hemoglobin	0.996 (0.983-1.010)	0.594			0.983 (0.969-0.997)	0.016*	0.993 (0.977-1.009)	0.365
PLT	1.000 (0.998-1.001)	0.536			1.001 (1.000-1.002)	0.108		
CRP	1.005 (1.002-1.009)	0.003*	1.003 (0.998-1.007)	0.229	1.002 (0.999-1.006)	0.167		
Albumin	0.927 (0.885-0.971)	0.001*^b^	0.937 (0.888-0.989)	0.019*	0.950 (0.910-0.993)	0.022*^c^		
LAR	1.067 (1.019-1.118)	0.006*^b^			1.023 (0.984-1.063)	0.257		
CSF WBC >500× 10^6^/L (CSF)	1.405 (0.852-2.318)	0.183			2.019 (1.228-3.319)	0.006*	1.742 (1.004-3.022)	0.048*
Glucose (CSF)	0.873 (0.749-1.018)	0.084			0.778 (0.666-0.908)	0.002*^c^		
Protein (CSF)	1.023 (0.987-1.060)	0.212			1.041 (0.993-1.091)	0.092		
Chloride (CSF)	0.975 (0.946-1.004)	0.090			0.947 (0.918-0.978)	<0.001*^c^ 0.001*=c		
Brain parenchymal damage	1.412 (0-821-2.431)	0.212			1.259 (0.733-2.161)	0.404		
Intracranial hemorrhage	0.978 (0.555-1.724)	0.938			1.969 (1.108-3.497)	0.021*^d^		
CSF culture (GNB)	1.268 (0.726-2.213)	0.404			3.991 (2.150-7.409)	< 0.001*	2.949 (1.517-5.732)	0.001*

LOS, Length of stay; WBC, White blood cell count; PLT, Platelet; CRP, C-reactive protein; LAR, Lactate dehydrogenase/Albumin; GNB, Gram-negative bacteria; Include the independent variables that had a *P* value < 0.05 but did not have a high correlation in the multivariate regression analysis. ^a, d^In Kendall correlation coefficient analysis, the associated variables showed a statistically significant correlation ^b^In Pearson correlation coefficient analysis, the associated variables showed a statistically significant correlation; ^c^In Pearson correlation coefficient analysis, age showed significant correlations with LOS, albumin, glucose and chloride in CSF; ^*^P < 0.05.

### Pathogens composition and distribution according to different groups

**Figure 1:**
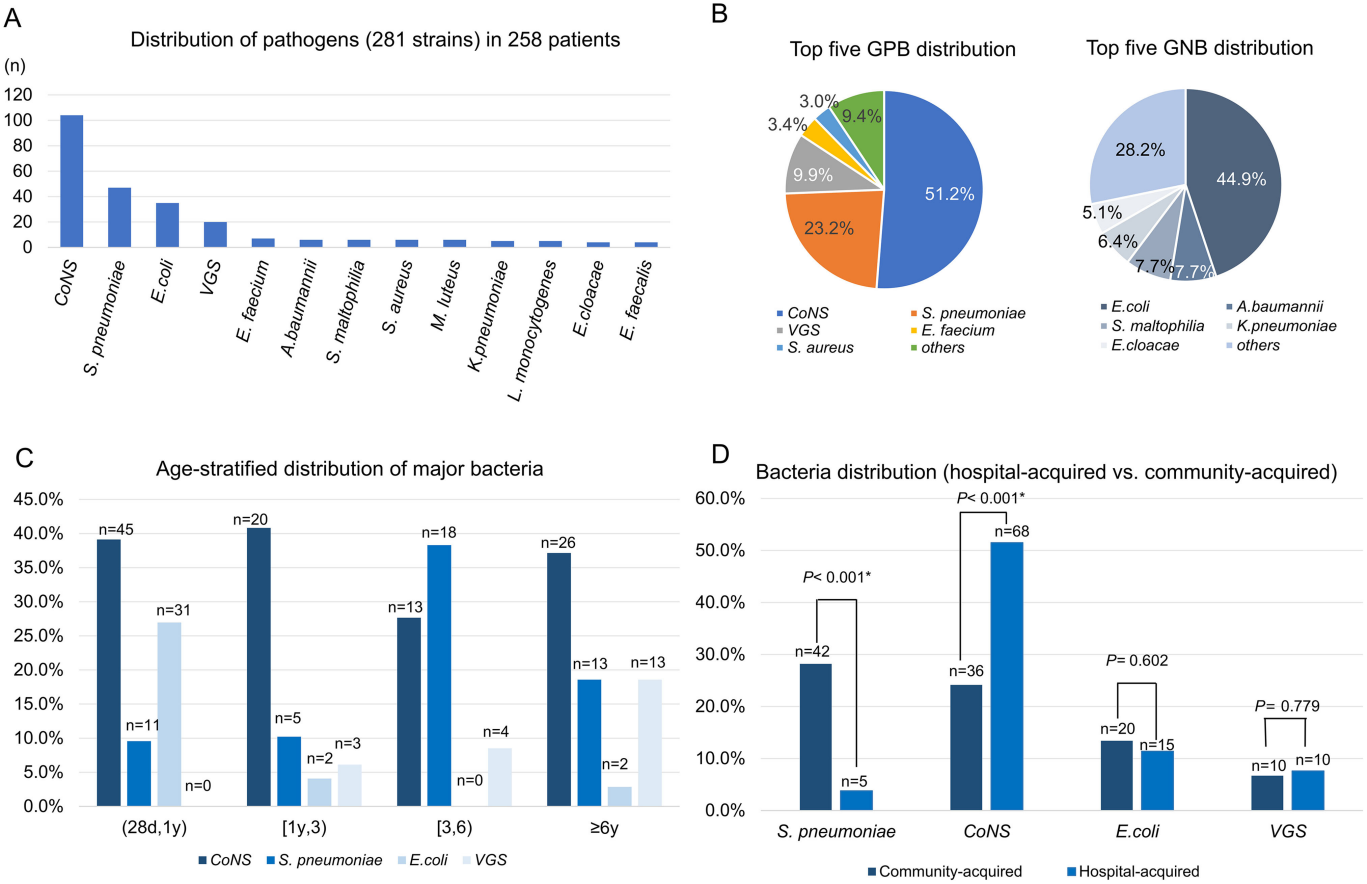
Frequency and distribution of bacteria in this study. **(A)** Distribution of pathogens in 258 patients. **(B)** Top five GPB and GNB distribution. **(C)** Distribution of bacteria in different age groups. **(D)** The distribution of bacteria between hospital-acquired and community-acquired PBM patients. *CoNS*, *Coagulase-negative staphylococci*; *S. pneumoniae*, *Streptococcus pneumoniae*; *E. coli*, *Escherichia coli*; *VGS*, *Viridans group streptococci*; *E. faecium*, *Enterococcus faecium*; *A. baumannii*, *Acinetobacter baumannii*; *S. maltophilia*, *Stenotrophomonas maltophilia*; *S. aureus*, *Staphylococcus aureus*; *M. luteus*, *Micrococcus luteus*; *K. pneumoniae*, *Klebsiella pneumoniae*; *L. monocytogenes*, *Listeria monocytogenes*; *E. cloacae*, *Enterobacter cloacae*; *E. faecalis*, *Enterococcus faecalis*; GPB, gram-positive bacteria; GNB, gram-negative bacteria; d, days; y, years; *p < 0.05.

During the study period, a total of 281 pathogenic bacteria were isolated from CSF cultures of 258 PBM cases. Among these, 203 strains (72.2%) were identified as GPB, and 78 strains (27.8%) were identified as GNB. Double organism growth was detected in 13 cultures and trio-organism growth was detected in 5 cultures. The most isolated pathogens were *coagulase-negative Staphylococcus* (CoNS), Streptococcus *pneumoniae* (*S. pneumoniae*) and *Escherichia coli* (*E.coli*) (**Figure 1A**). **Figure 1B** showed the top five bacteria in GPB and GNB, respectively. Among Gram-negative bacteria, *Acinetobacter baumannii* (*A. baumannii*) accounted for 7.7% (6/78) of isolates, representing 2.1% (6/281) of all positive cultures. The bacteria distribution varies by different ages, the detection rate of *E.coli* was the highest during infancy (27.0%), while the detection rate of *S. pneumoniae* was the highest among preschool children (38.3%) (**Figure 1C**). The distribution of bacteria changed before and after the COVID-19 pandemic (**Supplementary Figure 2A**). Specifically, CoNS represented 33.3%, 36.2%, and 39.6% of isolates before, during, and after the pandemic, respectively, while *S. pneumoniae* accounted for 29.2%, 14.2%, and 14.2% of isolates over the same periods. Patients were classified as community-acquired or hospital-acquired meningitis according to the criteria defined in the Methods. The microbial profiles differed markedly between them shown in **Figure 1D**. Among community-acquired, the most common pathogens were *S. pneumoniae* (28.2%), CoNS (24.2%), and *E.coli* (13.4%). In contrast, among hospital-acquired cases, the predominant organisms were CoNS (51.5%), *E.coli* (11.4%), and *Viridans group streptococci* (VGS) (7.6%). The detection rate of *S. pneumoniae* in hospital-acquired group is lower than that in community-acquired group, while the rate of CoNS is higher(). Compared with non-refractory group, the detection rate of *E.coli* in refractory group is higher (*P* < 0.05) (**Supplementary Figure 2B**).

### Antimicrobial resistance pattern of bacterial isolates in PBM

Among GPB, methicillin resistance was highly prevalent (MRCoNS 88.2%). Inducible clindamycin resistance (D-test positive) was detected in 57.8% of CoNS and 100% of *S. pneumoniae*. High-level macrolide resistance (erythromycin) was universal in *S. pneumoniae* (100%) and common in CoNS and *VGS*. No vancomycin- or linezolid-resistant Gram-positive isolates were found. Among GNB, ESBL production was identified in 54.3% of *E. coli* and 25.0% of *K. pneumoniae*. Carbapenem resistance was observed in 0.0% of *E. coli* and 40.0% of *K. pneumoniae*. *A. baumannii* isolates (n=6) were all hospital-acquired; 66.7% were carbapenem-resistant but remained susceptible to moxifloxacin and levofloxacin. Detailed resistance rates for individual antibiotics are provided in **Table 2**.

**Table 2:** Antimicrobial resistance rates of main bacteria isolated from patients with positive CSF cultures bacterial meningitis.

GPB	ERY(%)	LVX(%)	LZD(%)	TCY(%)	VAN(%)	SXT(%)	MXF(%)	CLI(%)	CHL(%)	PEN(%)	RIF(%)	Q/D(%)
* CoNS* (n=104)	75.5	47.1	0.0	41.5	0.0	30.4	24.5	57.8	0.0	90.9	10.8	0.0
* S.pneumoniae* (n=47)	100.0	2.1	0.0	90.9	0.0	63.8	0.0	100.0	7.0	66.7	0.0	NA
* VGS* (n=20)	78.9	10.5	0.0	58.8	0.0	NA	NA	73.7	6.3	10.0	NA	18.8
GNB	AMC(%)	AMP(%)	ATM(%)	ETP(%)	SXT(%)	GEN(%)	TGC(%)	FEP(%)	CRO(%)	LVX(%)	CAZ(%)	IPM(%)
* E.coli* (n=35)	5.7	87.5	26.1	0.0	71.4	25.0	0.0	22.9	58.8	68.6	14.3	0.0
* A.baumannii* (n=6)	100.0	100.0	100.0	100.0	16.7	66.7	0.0	33.3	40.0	16.7	50.0	50.0

GPB, Gram-positive bacteria; GNB, Gram-negative bacteria; ERY, Erythromycin; LVX, Levofloxacin; LZD, Linezolid; TCY, Tetracycline; VAN, Vancomycin; SXT, Trimethoprim-Sulfamethoxazole; MXF, Moxifloxacin; CLI, Clindamycin; CHL, Chloramphenicol; PEN, Penicillin; RIF, Rifampin; Q/D, Quinupristin/ Dalfopristin; AMC, Amoxicillin-clavulanic acid; AMP, Ampicillin; ATM, Aztreonam; ETP, Ertapenem; GEN, Gentamicin; TGC, Tigecycline; FEP, Cefepime; CRO, Ceftriaxone; CAZ, Ceftazidime; IPM, Imipenem; *CoNS*, *Coagulase-Negative Staphylococci*; *S.pneumoniae*, *Streptococcus pneumoniae*; *VGS*, *Viridans group streptococci*; *E.coli*, *Escherichia coli*; *A.baumannii*, *Acinetobacter baumannii*; NA, Not applicable.

### Comparison of bacterial resistance patterns across different groups

Antibiotic resistance patterns of major GPB and *E. coli* across different groups are summarized in **Supplementary Figure 3**. In descriptive comparisons, the antibiotic resistance profiles of CoNS and *S. pneumoniae* between ICU and non-ICU cases (**Supplementary Figure 3A**). The resistance rates of CoNS to levofloxacin (52.9% vs 32.3%) and trimethoprim-sulfamethoxazole (34.3% vs 22.9%) were higher in the hospital-acquired group, while that of erythromycin (85.3% vs 70.6%) and tetracycline (50.0% vs 39.4%) was higher in the community-acquired group (**Supplementary Figure 3B**). In the refractory group, CoNS showed a levofloxacin resistance rate of 57.5%; in the non-refractory group, the rate was 40.3%. For *S. pneumoniae*, the resistance rate to trimethoprim-sulfamethoxazole was 83.3% in the refractory group and 57.1% in the non-refractory group (**Supplementary Figure 3C**). There is not much difference in *E.coli* in antibiotic resistance among different groups (**Supplementary Figure 3D**).

### The distribution of special antimicrobial resistance phonotypes in major GNB

**Figure 2:**
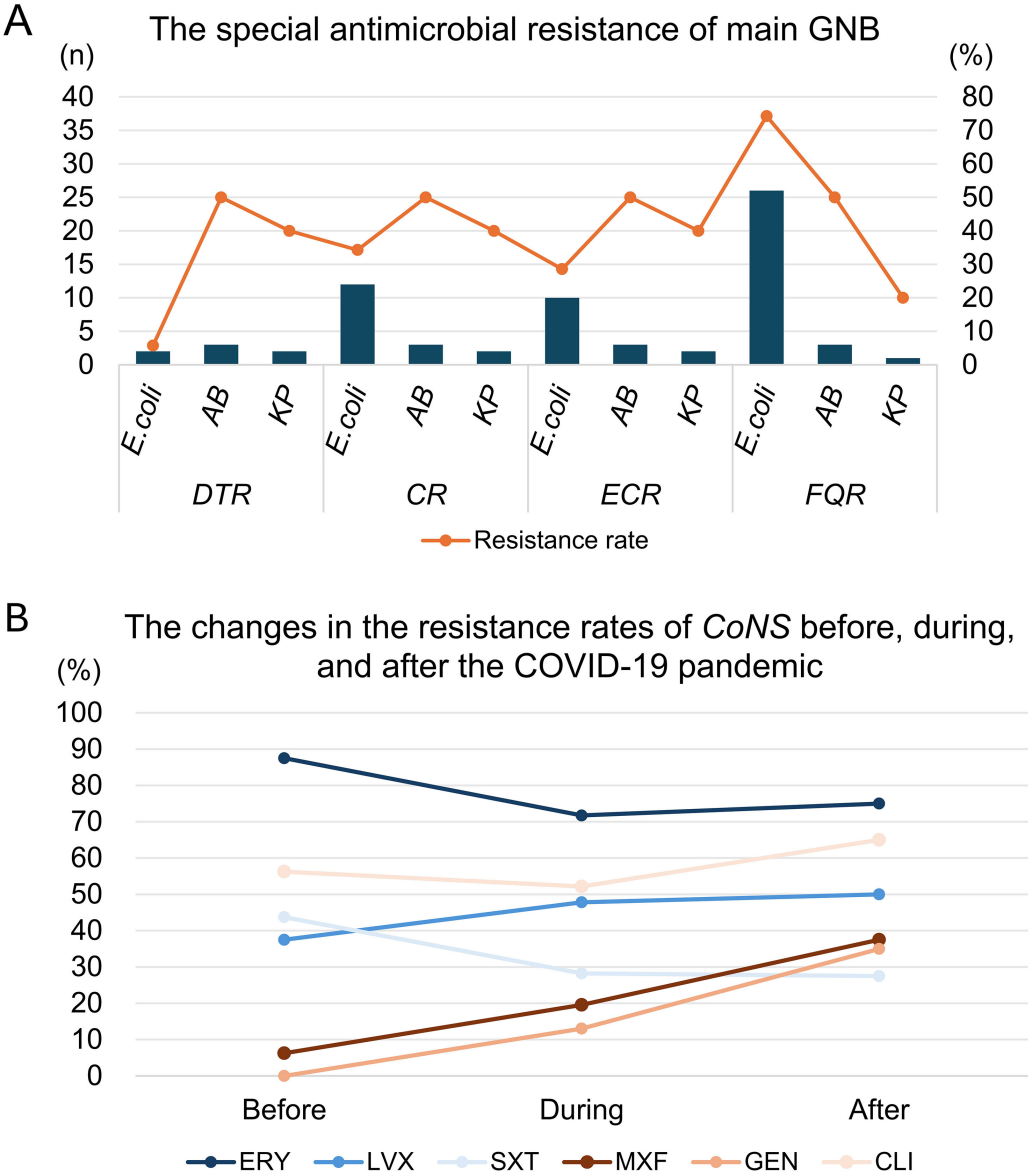
Analysis of special antibiotic resistance and changes in the detection rate of special bacteria. **(A)** The special antimicrobial resistance patterns of *E. coli*, *A. baumannii* and *K. pnumoniae*. **(B)** The changes in the resistance rates of *CoNS* according to the COVID-19 pandemic. DTR, difficult-to-treat resistance; CR, carbapenem resistance; ECR, extended-spectrum cephalosporin resistance; FQR, fluoroquinolone resistance; ERY, Erythromycin; LVX, Levofloxacin; SXT, Trimethoprim-Sulfamethoxazole; MXF, Moxifloxacin; GEN, Gentamicin; CLI, Clindamycin; *MRCoNS*, *Methicillin-Resistant Coagulase-Negative Staphylococci*; ESBL*- E.coli*, Extended- Spectrum β- Lactamase-producing *Escherichia coli*; *E.coli*, *Escherichia coli*; *AB, Acinetobacter baumannii*; *KP, Klebsiella pneumoniae*; GNB, gram-negative bacteria; Pre- COVID-19 pandemic (2018–09 to 2019-12), during (2020–01 to 2022-12), post- (2023–01 to 2025-08). Seven year-stages were divided from 2018–09 to 2025-08, for instance Year 1 represents 2018–09 to 2019-08, and so on.

As shown in **Figure 2A**, the detection rates of DTR, CR, ECR, and FQR were 5.7%, 34.3%, 28.6% and 74.3% in *E. coli*. The analysis results of the special drug resistance phenotypes of *E. coli*, *A. baumannii* and *K. pnumoniae* in this study indicated that FQR was the most predominant drug resistance phenotype. Among them, the detection rate of FQR (74.3%) in *E. coli* was the highest, while the DTR rate was the lowest (5.7%). Before, during and after the COVID-19 pandemic, the resistance rate of CoNS to moxifloxacin, gentamicin and levofloxacin showed an upward trend, while the resistance rate to trimethoprim-sulfamethoxazole showed a downward trend (**Figure 2B**). The overall detection rate of the MRCoNS isolates was approximately 88.2% and declined from 96.2% in 2019 to 80% in 2025 (**Supplementary Figure 4A**). The detection rate of ESBL-producing *E. coli* and fluctuated between 0% and 83.3% in 2019-2024 (**Supplementary Figure 4B**).

## Discussion

This 7-year retrospective study describes the bacterial spectrum and AMR profiles of pathogens isolated from CSF cultures in children with bacterial meningitis in Southwest China, with nearly half (43.0%) of the cohort being post-neurosurgical cases—a clinically distinct population whose microbiological findings should not be generalized to all pediatric meningitis in the region. Our findings reveal a persistently high burden of AMR and notable shifts in pathogen distribution compared to historical data, underscoring the need for localized surveillance.

It is important to emphasize that the epidemiology of bacterial meningitis differs fundamentally between healthcare-associated and community-acquired settings. In post-neurosurgical meningitis, CoNS and Gram-negative bacilli predominate, whereas community-acquired cases are typically caused by *S. pneumoniae*, *N. meningitidis*, and *Haemophilus influenzae*. The high prevalence of CoNS (37.0%) and MRCoNS (88.2%) in our cohort thus reflects the distinctive microbiology of post-neurosurgical infections rather than the general profile of pediatric meningitis in Southwest China.

In the present study, CoNS (37.0%) was the leading causative pathogen, followed by *S. pneumoniae* (16.7%) and *E.coli* (12.5%). Our research results are different from various reports from around the world, including those from the UK (Okike et al., 2014a), Africa (Agossou et al., 2019), and the United States (Castelblanco et al., 2014). All these reports indicate that *Neisseria meningitidis*, *S. pneumoniae*, and *Haemophilus influenzae* are the main pathogenic bacteria causing purulent meningitis. However, the results were similar to those of a multicenter study in 2016 to 2018 from China, which showed that the three leading pathogens causing PBM were *Staphylococcus epidermidis*, *E.coli* and *S. pneumoniae* (Peng et al., 2021). The reasons for the differences among the various studies are likely to be the variations in social and economic conditions as well as environmental factors in these regions. Therefore, it is urgent to promptly summarize and report the pathogenic characteristics of local pathogens.

Notably, 41.5% of all cases of PBM occur in children under the age of one, emphasizing the significant impact of this infection in this age group globally (Kim, 2010). The distribution of pathogens in children of different age groups varies greatly (Li et al., 2018; Peng et al., 2021). We noticed that in infants, the top 3 pathogens were CoNS (39.1%), *E. coli* (27.0%), and *S. pneumoniae* (9.6%). This result was not in accordance with several studies from the USA, the UK and Ireland (Okike et al., 2014b; Woll et al., 2018), which showed that group B Streptococcus, *E. coli* and *Staphylococcus aureus* were the predominant pathogens causing PBM in infants. Meanwhile, this result was not consistent with study from Southern China (Chen et al., 2025), which showed the main pathogens were *E. coli*, *Streptococcus agalactiae* and *S. pneumoniae.* We also observed that in children older than one year of age, the predominant organisms causing PBM were CoNS, *S. pneumoniae*, and *E. coli* (27.0%). This finding was not consistent with a previous study conducted in Southern China, with predominant of *S. pneumoniae* (Chen et al., 2025). The prevalence of *S. pneumoniae* in our cohort should be considered in the context of local pneumococcal conjugate vaccine (PCV) coverage. In China, PCV13 is a voluntary vaccine and its uptake is suboptimal, with coverage rates significantly lower than the global average. The most common pneumococcal serotypes causing pediatric invasive disease in China (e.g., 19F, 14, 23F, 6B, 19A) are covered by PCV13, highlighting the potential impact of vaccination on pathogen distribution (Liu et al., 2023). The low vaccine coverage observed nationally likely contributes to the continued clinical significance of *S. pneumoniae* as a major pathogen in pediatric meningitis (Ning et al., 2024). Therefore, for patients with PBM, the selection of empirical antibacterial drugs should be classified according to age to cover the most likely pathogens.

The pathogen spectrum of PBM in Southwest China showed significant shifts during and after the COVID-19 pandemic. The detection rate of *S. pneumoniae* declined markedly from 29.2% pre-pandemic to 14.2% during the pandemic and remained at this lower level thereafter, likely due to non-pharmaceutical interventions (e.g., masking, social distancing) that reduced respiratory pathogen transmission, as well as changes in healthcare-seeking behavior. In contrast, CoNS showed a continuous upward trend, reaching 39.6% post-pandemic, which may reflect the increasing proportion of healthcare-associated infections in this cohort. To our knowledge, few studies have reported longitudinal trends of CoNS in PBM. Compared with a retrospective study including 835 pediatric cases by Zhang et al. (2024b) from 2017 to 2023 in Shandong province showing CoNS represented 38.6%, 36.1%, and 19.8% of isolates before, during, and after the pandemic, respectively. Our finding of a continuous rise to 39.6% may be explained by a higher frequency of neurosurgical procedures and limited sample size in our cohort. Interestingly, this pattern differs from a previous study (Zhang et al., 2024b) reporting a significant decrease in CoNS and a notable increase in *E. coli* after COVID-19, suggesting that the impact of the pandemic on bacterial pathogen distribution may vary by region, healthcare setting, and patient population.

In our cohort, *S. pneumoniae* and *E. coli* remained the dominant pathogens in community-acquired bacterial meningitis, consistent with global trends (Collaborators, G.B.D.M.A.R, 2023). Age-stratified analysis reinforced the classic paradigm: *E. coli* predominated in infants, while *S. pneumoniae* became more prevalent in older children. Of particular concern was the high level of antimicrobial resistance observed in *E. coli*. The detection rate of ESBL-producing *E. coli* fluctuated widely (0%–83.3%) over the study period, suggesting possible clonal outbreaks or variable infection control practices, and highlighting the unreliability of assuming stable resistance patterns. Conversely, the relatively low rate of difficult-to-treat resistance (5.7%) in *E. coli* provides reassurance regarding the potential effectiveness of carbapenems and other last-line antibiotics.

In hospital-acquired cases, CoNS were significantly more common than in community-acquired infections. CoNS was the most frequently isolated organism, a finding consistent with some recent reports but higher than traditional epidemiological data for community-acquired meningitis (Vena et al., 2013; Zheng et al., 2020; van Ettekoven et al., 2024). This high isolation rate, coupled with the fact that 43.0% of our patients underwent neurosurgical procedures and the alarmingly high methicillin resistance rate among CoNS (88.2%, confirmed by cefoxitin disk diffusion), presents a clinical dilemma. While often dismissed as contaminants, in a cohort with a high rate of post-neurosurgical status, a significant proportion of these CoNS isolates likely represent true healthcare-associated infections, particularly associated with external ventricular drains or shunts (Tunkel et al., 2017). The high MRCoNS rate effectively renders empirical beta-lactam antibiotics (like oxacillin or first-generation cephalosporins) ineffective in this subset of patients. Therefore, for children with risk factors like prior neurosurgery or indwelling devices, empirical therapy covering MRCoNS (e.g., vancomycin) may be warranted while culture results are pending, provided that diagnostic confirmation is vigorously pursued to avoid unnecessary antibiotic exposure. However, differentiating true infection from contamination remains a critical challenge that requires correlation with clinical presentation and CSF parameters (Akram Asif et al., 2023; AbdelHalim et al., 2025).

The isolation of *A. baumannii* and *K. pneumoniae*, together with polymicrobial growth in 18 cultures (7.0% of cases), is noteworthy. These findings are strongly associated with healthcare-associated infections, particularly in patients with prolonged hospitalization, prior antibiotic exposure, or neurosurgical devices (Chen et al., 2020) The presence of multi-drug resistant (MDR) GNB like *A. baumannii* poses a significant therapeutic challenge, as treatment options are often limited to combinations of agents like ampicillin/sulbactam, carbapenems, or tigecycline, whose CNS penetration and efficacy can be variable (Kumar et al., 2021). The high rate of complications such as hydrocephalus (26.4%) and subdural effusion (24.4%) in our cohort may be partly attributable to the severity of inflammation induced by these MDR organisms.

Our data provides contemporary, local quantification of the resistance burden that clinicians face when applying existing guidelines. For community-acquired meningitis, a third-generation cephalosporin plus vancomycin remains the standard recommended regimen (Luo et al., 2023). However, in neonates and infants in regions with a high prevalence of ESBL-producing *E. coli*, early consideration of a carbapenem is reasonable if Gram-negative rods are seen on CSF Gram stain or if the child is critically ill with ESBL risk factors. For hospital-acquired (post-neurosurgical) meningitis, the IDSA-recommended regimen of vancomycin plus an anti-pseudomonal agent (e.g., cefepime, meropenem) is strongly supported by our finding of high MRCoNS (88.2%) and MDR Gram-negative bacilli (Luo et al., 2023). This study does not call for novel empirical regimens but rather validates the continued appropriateness of these established standards in our pediatric neurosurgical population, while highlighting the urgent need for enhanced diagnostic confirmation and antimicrobial stewardship.

## Limitations

This study has several limitations. First, the single-center design and the high proportion of post-neurosurgical cases (43.0%) limit the generalizability of our findings to community-acquired pediatric meningitis or to other regions with different healthcare settings. Second, the lack of a consensus definition of “refractory bacterial meningitis” limits direct comparisons across studies, and the criteria used here should be interpreted as an exploratory subgroup classification rather than a validated clinical entity. Future multicenter studies should adopt consistent definitions to validate our findings. Third, reliance on CSF culture may underestimate the true burden of pathogens, especially in cases where prior antibiotics were administered. The use of molecular diagnostic techniques would provide a more complete picture. Fourth, we were unable to differentiate definitively between CSF contamination and true infection for CoNS, which may have led to an overestimation of their role as true pathogens. Fifth, detailed clinical outcomes and antibiotic dosing information were not systematically analyzed to establish direct correlations with specific resistance patterns. Finally, this study only included culture-positive cases of bacterial meningitis. Culture-negative meningitis, which may account for a substantial proportion of pediatric bacterial meningitis, particularly when antibiotics are administered prior to lumbar puncture, was not captured.

## Conclusion

In conclusion, this study provides local, contemporary data on pediatric post-neurosurgical bacterial meningitis in Southwest China (2018–2025). It quantifies the high burden of MRCoNS (88.2%), ESBL-producing *E. coli* (31.4%), and carbapenem-resistant *A. baumannii* (66.7%), validating the continued appropriateness of IDSA-recommended risk-stratified empirical regimens. Continuous local surveillance and antimicrobial stewardship remain essential.

## Data Availability

The original contributions presented in the study are included in the article/**Supplementary Material**. Further inquiries can be directed to the corresponding authors.

## Supplementary material

Supplementary Figure 1 Flowchart of patients’ enrollment and study design in positive CSF pediatric bacterial meningitis.

Supplementary Figure 2 **(A)** Distribution of major bacterial species pre-, during, and post-COVID-19 pandemic. **(B)** The distribution of bacteria between refractory and non-refractory groups. Pre-pandemic, September 1, 2018 to December 31, 2019; During the pandemic, January 1, 2020 to December 31, 2022; Post-pandemic, January 1, 2023 to September 30, 2025.

Supplementary Figure 3 Antimicrobial resistance rates among different groups. **(A–C)** Resistance rates comparison among major GPB between ICU and non-ICU, surgery and non-surgery, refractory and non-refractory. **(D)** Resistance rates comparison of *E.coli* between ICU and non-ICU, surgery and non-surgery, refractory and non-refractory. ERY, Erythromycin; LVX, Levofloxacin; LZD, Linezolid; TCY, Tetracycline; VAN, Vancomycin; SXT, Trimethoprim-Sulfamethoxazole; MXF, Moxifloxacin; AMC, Amoxicillin-clavulanic acid; AMP, Ampicillin; ATM, Aztreonam; ETP, Ertapenem; GEN, Gentamicin; TGC, Tigecycline; FEP, Cefepime; CAZ, Ceftazidime; IPM, Imipenem; *CoNS*, *Coagulase-Negative Staphylococci*; *S.pneumoniae*, *Streptococcus pneumoniae*; *E.coli*, *Escherichia coli*; GPB, Gram-positive bacteria.

Supplementary Figure 4 Annual distribution of *MRCoNS* and ESBL*-E.coli*.
