# Supplementary figures and images for: Bacterial spectrum and antimicrobial resistance of cerebrospinal fluid pathogens in pediatric bacterial meningitis: a 7-year study in Southwest China with emphasis on post-neurosurgical cases
Source: Front Cell Infect Microbiol. 2026 Jul 10;16:1842744. doi: 10.3389/fcimb.2026.1842744 (PMC13395615; doi:10.3389/fcimb.2026.1842744)

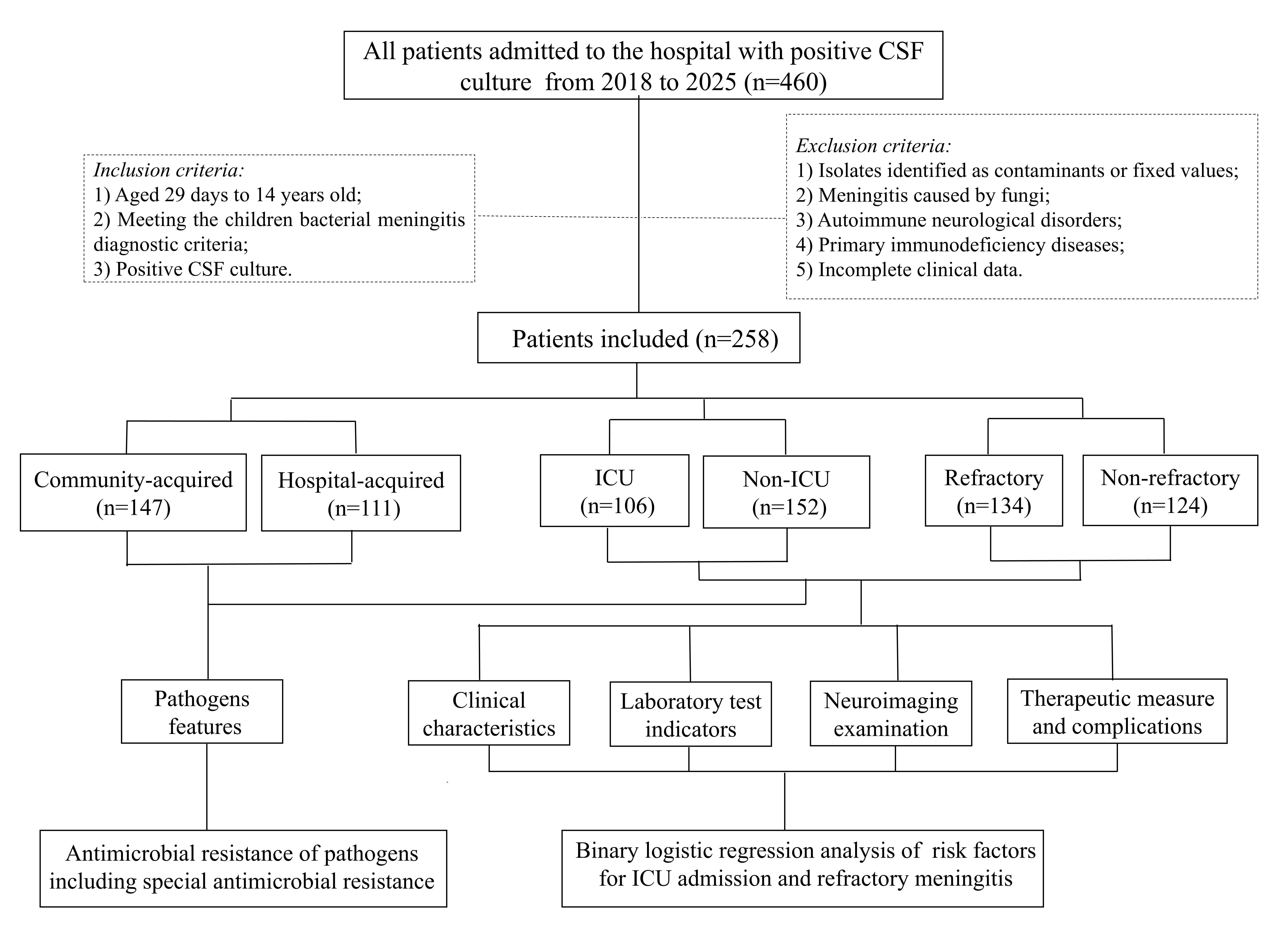

Supplement: Supplementary Figure 1 — Flowchart of patients’ enrollment and study design in positive CSF pediatric bacterial meningitis. [file Image1.jpeg]
