# Supplementary material for: Bacterial spectrum and antimicrobial resistance of cerebrospinal fluid pathogens in pediatric bacterial meningitis: a 7-year study in Southwest China with emphasis on post-neurosurgical cases
Source: Front Cell Infect Microbiol. 2026 Jul 10;16:1842744. doi: 10.3389/fcimb.2026.1842744 (PMC13395615; doi:10.3389/fcimb.2026.1842744)
Supplement: Supplementary file 5 [file Table1.docx]

| Table S1. Clinical features of children with CSF culture positive -bacterial meningitis. | | | | | | | | | |
| --- | --- | --- | --- | --- | --- | --- | --- | --- | --- |
| Items | All patients (n=258） | Group 1 | | |  | Group 2 | | | |
|  |  | ICU (n=106） | Non-ICU (n=152） | *P* |  | Refractory (n=134） | Non-refractory (n=124) | | *P* |
| Male, n (%) | 150 (58.1) | 57 (54.3) | 93 (60.8) | 0.299 |  | 77 (57.5) | 73 (58.9) | 0.819 | |
| Age (mo) | 17.0 (4.8, 64.0) | 10.0 (3.0, 61.0) | 25.5 (7.0, 72.0) | 0.006* |  | 10.4 (4.0, 48.0) | 37.5 (7.1, 81) | ＜0.001* | |
| LOS (days) | 28.0 (18.0, 41.0) | 24.0 (13.5, 39.0) | 29.5 (21.0, 42.0) | 0.020* |  | 33.0 (24.0, 43.0) | 22.0 (15.0, 35.8) | ＜0.001* | |
| Preterm birth , n (%) | 24 (9.3) | 9 (8.6) | 15 (9.8) | 0.738 |  | 17 (12.7) | 7 (5.6) | 0.052 | |
| Low birth weight, n (%) | 31 (12.0) | 16 (15.2) | 15 (9.8) | 0.187 |  | 19 (14.2) | 12 (9.7) | 0.267 | |
| Surgery, n (%) | 111 (43.0) | 34 (32.4) | 77 (50.3) | 0.004* |  | 72 (53.7) | 39 (31.5) | ＜0.001* | |
| Symptoms/Signs, n (%) |  |  |  |  |  |  |  |  | |
| Fever, n (%) | 219 (84.9) | 94 (89.5) | 125 (81.7) | 0.085 |  | 114 (85.1) | 105 (84.7) | 0.929 | |
| Seizure, n (%) | 58 (22.5) | 31 (29.5) | 27 (17.6) | 0.025* |  | 36 (26.9) | 22 (17.7) | 0.079 | |
| Vomiting, n (%) | 95 (36.8) | 39 (37.1) | 56 (36.6) | 0.929 |  | 42 (31.3) | 53 (42.7) | 0.058 | |
| Lethargy/Coma, n (%) | 64 (24.8) | 31 (29.5) | 33 (21.6) | 0.146 |  | 33 (24.6) | 31 (25.0) | 0.945 | |
| Nuchal rigidity, n (%) | 77 (29.8) | 37 (35.2) | 40 (26.1) | 0.117 |  | 34 (25.4) | 43 (34.7) | 0.013* | |
| Babinski sign, n (%) | 82 (31.8) | 42 (40) | 40 (26.1) | 0.019* |  | 44 (32.8) | 38 (30.6) | 0.706 | |
| Hematological examination |  |  |  |  |  |  |  |  | |
| WBC (4.0-10.0×10⁹/L) | 11.4 (8.3, 17.2) | 13.8 (8.7, 19.2) | 10.4 (8.1, 14.6) | 0.022* |  | 12.4 (8.6, 16.5) | 10.6 (8.1, 18.4) | 0.653 | |
| NC (1.0-6.0×10⁹/L) | 7.8 (4.2, 11.7) | 8.4 (4.7, 14.1) | 7.1 (3.7, 10.8) | 0.057 |  | 7.8 (4.2, 11.7) | 7.7 (3.4, 11.7) | 0.879 | |
| LC (2.0-9.0×10⁹/L) | 2.5 (1.6, 4.1) | 2.7 (1.8, 4.4) | 2.4 (1.6, 3.9) | 0.196 |  | 2.7 (1.7, 4.2) | 2.4 (1.5, 4.0) | 0.317 | |
| Hb (110-140g/L) | 111.6 ± 17.9 | 110.1 ± 20.9 | 112.5 ± 15.5 | 0.613 |  | 109.2 ± 15.8 | 114.2 ± 19.7 | 0.015* | |
| PLT (100-300×10^⁹/L) | 351.0 (256.0, 506.0) | 353.0 (254.5, 524.0) | 343.0（258.3, 473.3） | 0.635 |  | 369.0 (267.0, 537.0) | 326.0 (253.3, 429.5) | 0.048* | |
| CRP (0-10mg/L) | 41.9 (5.0, 121.1) | 71.1 (17.0, 179.6) | 28.8 (3.3, 95.5) | 0.002* |  | 56.4 (8.0, 134.3) | 33.6 (3.6, 112.0) | 0.075 | |
| LDH (140-280U/L) | 269.0 (220.5, 355.0) | 298.6 (235.7, 361.5) | 254.0 (210.8, 336.3) | 0.003* |  | 267.0 (220.5, 355.0) | 271.0 (219.1, 358.5) | 0.963 | |
| Albumin (39-54g/L) | 34.7 ± 5.7 | 33.3 ± 5.7 | 35.7 ± 5.5 | ＜0.001* |  | 33.8 ± 5.4 | 35.7 ± 5.9 | 0.021* | |
| LAR | 7.68 (6.14, 10.63) | 8.74 (6.72, 11.27) | 6.93 (5.94, 9.) | ＜0.001* |  | 7.88 (6.25, 11.01) | 7.53 (5.90, 10.07) | 0.274 | |
| MLR | 0.28 (0.17, 0.48) | 0.28 (0.18, 0.45) | 0.28 (0.16, 0.50) | 0.997 |  | 0.32 (0.20, 0.52） | 0.24 (0.13, 0.46) | 0.071 | |
| NLR | 2.78 (1.20, 6.66) | 2.58 (1.36, 6.90) | 2.93 (1.11, 6.41) | 0.745 |  | 2.58 (1.26, 6.27) | 2.99 (1.14, 6.90) | 0.622 | |
| CSF test results |  |  |  |  |  |  |  |  | |
| WBC (×10^6^/L) | 598.0 (121.0, 3423.0) | 936.0 (168.5, 4508.5) | 408.5 (104.3, 2802.3) | 0.165 |  | 936.0 (240.0, 4491.0) | 349.5 (68.3, 2787.0) | ＜0.001* | |
| Glucose (2.8-4.5mmol/L) | 2.3 (0.6, 3.3) | 1.8 (0.5, 3.1) | 2.4 (0.6, 3.5) | 0.121 |  | 1.9 (0.5, 2.9) | 2.6 (1.1, 3.7) | ＜0.001* | |
| Protein (0-0.4g/L) | 0.85 (0.31, 1.96) | 1.22 (0.47, 2.35) | 0.73 (0.25, 1.90) | 0.002* |  | 1.22 (0.48, 2.40) | 0.63 (0.25, 1.52） | ＜0.001* | |
| Chloride (111-123mmol/L) | 120.1 (114.7, 125.0) | 119.7 (113.5, 124.2) | 120.3 (115.5, 125.6) | 0.131 |  | 119.1 (113.0, 123.8) | 122.2 (116.4, 126.6) | ＜0.001* | |
| Imaging data, n (%) |  |  |  |  |  |  |  |  | |
| Brain parenchymal damage | 75 (29.1) | 35 (33.3) | 40 (26.1) | 0.212 |  | 42 (31.3) | 33 (26.6) | 0.403 | |
| Cerebral softening | 35 (13.6) | 9 (8.6) | 26 (17.0) | 0.052 |  | 21 (15.7) | 14 (11.3) | 0.305 | |
| Intracranial hemorrhage | 67 (26.0) | 27 (25.7) | 40 (26.1) | 0.938 |  | 43 (32.1) | 24 (19.4) | 0.020* | |
| CSF culture (GNB), n (%) | 69 (26.7) | 31 (29.5) | 38 (24.8) | 0.403 |  | 52 (38.8) | 17 (13.7) | ＜0.001* | |
| Complications, n (%) |  |  |  |  |  |  |  |  | |
| Hydrocephalus | 68 (26.4) | 40 (26.1) | 28 (26.7) | 0.925 |  | 36 (26.9) | 32 (25.8) | 0.847 | |
| Subdural effusion/ empyema | 63 (24.4) | 27 (25.7) | 36 (23.5) | 0.688 |  | 63 (47.0) | 0 (0) | ＜0.001* | |
| Cerebral abscess | 16 (6.2) | 10 (9.5) | 6 (3.9) | 0.067 |  | 11 (8.2) | 5 (4) | 0.165 | |
| Treatments, n (%) |  |  |  |  |  |  |  |  | |
| IVIG | 39 (15.1) | 21 (20.0) | 18 (11.8) | 0.070 |  | 15 (11.2) | 24 (19.4) | 0.068 | |
| Corticosteroids | 179 (69.4) | 77 (73.3) | 102 (66.7) | 0.254 |  | 95 (70.9) | 84 (67.7) | 0.583 | |
| Mechanical ventilation | 72 (27.9) | 27 (25.7) | 45 (29.4) | 0.515 |  | 39 (29.1) | 33 (26.6) | 0.656 | |

Note. CSF, Cerebrospinal fluid; LOS, Length of stay; WBC, White blood cell count; NC, Neutrophil count; LC, Lymphocyte count; Hb, Hemoglobin; CRP, C-reactive protein; LDH,lactate dehydrogenase; LAR, Lactate dehydrogenase/ Albumin; MLR, Monocytel count/ Lymphocyte count; NLR, Neutrophil/ lymphocyte; GNB, Gram-negative bacteria; IVIG, Intravenous immunoglobulin; Preterm birth, Newborns with a gestational age of less than 37 weeks; Low birth weight, Newborns with a birth weight of less than 2500 grams; ^*^, *P* < 0.05.
